# Relationships between Habitual Polyphenol Consumption and Gut Microbiota in the INCLD Health Cohort

**DOI:** 10.3390/nu16060773

**Published:** 2024-03-08

**Authors:** Alexandra Adorno Vita, Kristen M. Roberts, Anders Gundersen, Yuliya Farris, Heather Zwickey, Ryan Bradley, Tiffany L. Weir

**Affiliations:** 1 Helfgott Research Institute, National University of Natural Medicine, Portland, OR 97201, USA; 2 Department of Food Science and Human Nutrition, Colorado State University, Fort Collins, CO 80523, USA; 3 School of Health and Rehabilitation Sciences, Ohio State University, Columbus, OH 43210, USA; 4 Pacific Northwest National Laboratory, Biological Sciences Division, Richland, WA 99352, USA; 5 Herbert Wertheim School of Public Health, University of California, San Diego, CA 92037, USA

**Keywords:** microbiome, polyphenols, culinary herbs and spices, phytochemicals

## Abstract

While polyphenol consumption is often associated with an increased abundance of beneficial microbes and decreased opportunistic pathogens, these relationships are not completely described for polyphenols consumed via habitual diet, including culinary herb and spice consumption. This analysis of the International Cohort on Lifestyle Determinants of Health (INCLD Health) cohort uses a dietary questionnaire and 16s microbiome data to examine relationships between habitual polyphenol consumption and gut microbiota in healthy adults (n = 96). In this exploratory analysis, microbial taxa, but not diversity measures, differed by levels of dietary polyphenol consumption. Taxa identified as exploratory biomarkers of daily polyphenol consumption (mg/day) included *Lactobacillus*, *Bacteroides*, *Enterococcus*, *Eubacterium ventriosum group*, *Ruminococcus torques group*, and *Sutterella.* Taxa identified as exploratory biomarkers of the frequency of polyphenol-weighted herb and spice use included *Lachnospiraceae UCG-001*, *Lachnospiraceae UCG-004*, *Methanobrevibacter*, *Lachnoclostridium*, and *Lachnotalea.* Several of the differentiating taxa carry out activities important for human health, although out of these taxa, those with previously described pro-inflammatory qualities in certain contexts displayed inverse relationships with polyphenol consumption. Our results suggest that higher quantities of habitual polyphenol consumption *may* support an intestinal environment where opportunistic and pro-inflammatory bacteria are represented in a lower relative abundance compared to those with less potentially virulent qualities.

## 1. Introduction

Polyphenols are phytochemicals present in various fruits, vegetables, nuts, beverages, and culinary herbs and spices in the diet [1], and evidence suggests that certain diet-derived phytochemicals (i.e., secondary plant metabolites), such as polyphenols, may beneficially modulate gut microbiota and mediate other clinically relevant biological outcomes through both microbiota-independent and microbiota-dependent mechanisms [2] Independent of interactions with microbiota, polyphenols exert direct effects through cell signaling, free-radical scavenging, and modulating gene expression and molecule production, thus altering cellular activity [3,4,5,6]. Polyphenol bioactivity is also mediated, at least in part, through interactions with gut microbiota [7,8]. Microbial metabolism of polyphenols results in bioactive metabolites that exert physiological effects both systemically and locally at the intestinal mucosa [9,10]. Taken together, evidence suggests these mechanisms beneficially impact microbial community structure [11,12,13,14], intestinal permeability [15,16], oxidative stress [4,9,17], and inflammatory [9,18,19,20], neurological [21,22,23], and cardiometabolic [24,25] processes.

While polyphenol consumption is suggested to be associated with an increased abundance of beneficial bacteria and a decrease in opportunistic and pro-inflammatory bacteria in certain contexts [13,14], evidence regarding the impact of polyphenol consumption on the abundance of specific taxa, microbial diversity, and functional communities is not consistent. It is worth noting that variability in an individual’s cardiometabolic health, substance use (e.g., smoking and alcohol use), exercise habits, healthy history, and several other factors may be reflected in variable gut microbial communities [26,27,28,29,30] and subsequent interactions with dietary polyphenols. Moreover, although the impacts of specific polyphenol-based interventions have been described in some detail, the impacts are not well described for polyphenols consumed via regular dietary habits, and consideration for the consumption of herbs and spices used in culinary settings is even more limited. Further investigating these relationships in the context of regular dietary habits in a cohort of generally healthy adults will provide insight into how habitual polyphenol consumption may support a gut microbial environment that facilitates health maintenance and, subsequently, disease prevention. 

In our previous research exploring associations between culinary herb and spice use and gut microbial taxa and diversity, we identified that the frequency of culinary herb and spice use was associated with microbial taxa at the phylum level, particularly regarding herbs and spices high in polyphenol content [31]. The current study aims to build on these previous findings by using data from the same cohort to (1) explore relationships between habitual dietary polyphenol consumption from food and beverage sources and microbial taxa and diversity, and (2) further explore relationships between the frequency of polyphenol-weighted herb and spice use and microbial taxa and diversity in healthy adults. To achieve this, we first explored potential microbial biomarkers of polyphenol exposure and then used these identified biomarkers in more targeted statistical comparisons. The findings of this research further inform our understanding of the relationship between habitual dietary patterns on microbiota and may inform future guidance on dietary intake of polyphenols via diet. 

## 2. Materials and Methods

### 2.1. Study Design and Participants

This study is a secondary analysis of the International Cohort on Lifestyle Determinants of Health (INCLD Health) cohort data, the methods of which have been previously described [32]. While the INCLD Health longitudinal cohort study collected data on various aspects of health and wellness, diet, and the gut microbiome over several time points at 6-month intervals, this secondary analysis only includes data from the baseline visit analyzed in a cross-sectional manner. From the original sample, a subsample (n = 96) was selected for this secondary analysis based on specific criteria. Inclusion criteria consisted of the full completion of all survey questions and data collection methods utilized for this secondary analysis, including data from the Demographic Questionnaire, Health History Questionnaire, Herb and Spice Frequency Questionnaire, VioScreen dietary analysis tool [33,34], and 16s rRNA microbiota analysis. Exclusion criteria consisted of past or current inflammatory bowel syndrome, inflammatory bowel disease (ulcerative colitis or Crohn’s disease), or celiac disease; past or current autoimmune disease; and current antibiotic use.

### 2.2. 16S rRNA Gene Sequencing and Processing

All 16sRNA gene sequencing and processing was performed by the Pacific Northwest National Laboratory (Richland, WA, USA). The Quick-DNA Fecal/Soil Microbe Microprep Kit (Zymo, Irvine, CA, USA) was used to extract microbial DNA from participant fecal samples. An Illumina MiSeq was used to sequence the hypervariable V4 region of the 16S rRNA gene using the 515F-806R primer set. The resulting 16S rRNA amplicon dataset was processed using QIIME2 (v2021.4) [35]. The DADA2 (*q2-dada2*) [36] pipeline within QIIME2 was used to denoise and cluster amplicon sequence variants (ASVs), which were then taxonomically classified (*q2-feature-classifer*) using the SILVA database (v138) [37]. Processed data were exported from QIIME2 and converted into a comma-delimited file.

### 2.3. Microbiome Data Filtering and Normalization

The data exported from QIIME2 were filtered and normalized in the MicrobiomeAnalyst online platform [38]. To remove features that may be a result of sequencing error or low-level contamination, a Low Count Filter removed reads with less than 4 counts and read that were present in less than 20% of the samples. To remove features that are close to constant throughout the experimental conditions and thus are not likely to be associated with the study conditions, a Low Variance Filter was applied to remove reads with less than 10% variance across samples, determined based on the interquartile range (IQR). Data were rarefied to minimum counts due to the large range in library sizes and data was scaled using total sum scaling. No data transformation was performed. 

### 2.4. Polyphenol Estimations from Vioscreen Dietary Data

To quantitate polyphenol intake from the VioScreen food frequency questionnaire (FFQ) output [33,34], an Excel spreadsheet was developed to catalog all foods in which participants identified in the FFQ. All mixed dishes were deconstructed to obtain individual foods by using the Food Commodity Intake Database (FCID, https://fcid.foodrisk.org/recipes/, accessed on 7 July 2023). All deconstructed recipes were reviewed by a team of research dietitians. All foods presumed to have minimal polyphenol content were removed from analysis. When foods were grouped together on the FFQ, individual foods comprising the group were weighted using 2005–2018 National Health and Nutrition Examination Survey (NHANES) data. The remaining foods were then matched to foods and beverages in the Phenol-Explorer database (PED) version 3.6 [1,39]. The PED contains data related to 501 individual polyphenols, which are further categorized into 18 sub-classes within the following 5 major classes: flavonoids, phenolic acids, lignans, stilbenes, and “other”. The flavonoids group contains 279 polyphenols; the phenolic acids group contains 108 polyphenols; the stilbenes group contains 10 polyphenols; the lignans group contains 29 polyphenols; and the “other” group contains 80 polyphenols. Content values in the PED were chosen based on the appropriate method for the food matrix and/or polyphenol subclass following previously published methods [40,41,42]. Retention factors were not applied. 

### 2.5. Exposure Variables

There are two main exposure variables: estimated dietary polyphenol consumption and frequency of polyphenol-weighted herb and spice use.

The *estimated dietary polyphenol consumption* variable is used both continuously and categorically. As a continuous variable, it is defined as the total estimated milligrams of polyphenols consumed per day (mg/day) from dietary food and beverage sources other than herbs and spices. As a categorical variable, participants are stratified into *low-*, *medium-*, or *high-consumer* groups based on the tertile distributions of the total estimated polyphenols consumed per day (mg/day). These tertile distributions are determined by the mg/day value of polyphenols consumed. 

Since the Vioscreen dietary analysis tool does not account for the consumption of individual herbs and spices, which may be high in polyphenols, the *frequency of polyphenol-weighted herb and spice use* categorical variable allows us to explore relationships that these polyphenol-rich sources may have with gut microbiota. To create this polyphenol-weighted frequency variable, the frequency of use of each herb and spice was first reported by participants and scored as follows: never (Score 0), once per month (Score 1), two-to-three times per month (Score 2), once per week (Score 3), two-to-three times per week (Score 4), three-to-four times per week (Score 5), five-to-six times per week (Score 6), or daily (Score 7). Then, we stratified participants into groups of *low-*, *medium-*, and *high-frequency* users of polyphenol-weighted herbs and spices based on the tertile distributions of calculated polyphenol-weighted frequency scores. These tertile distributions are determined by the value of frequency scores. 

**Figure 1:**
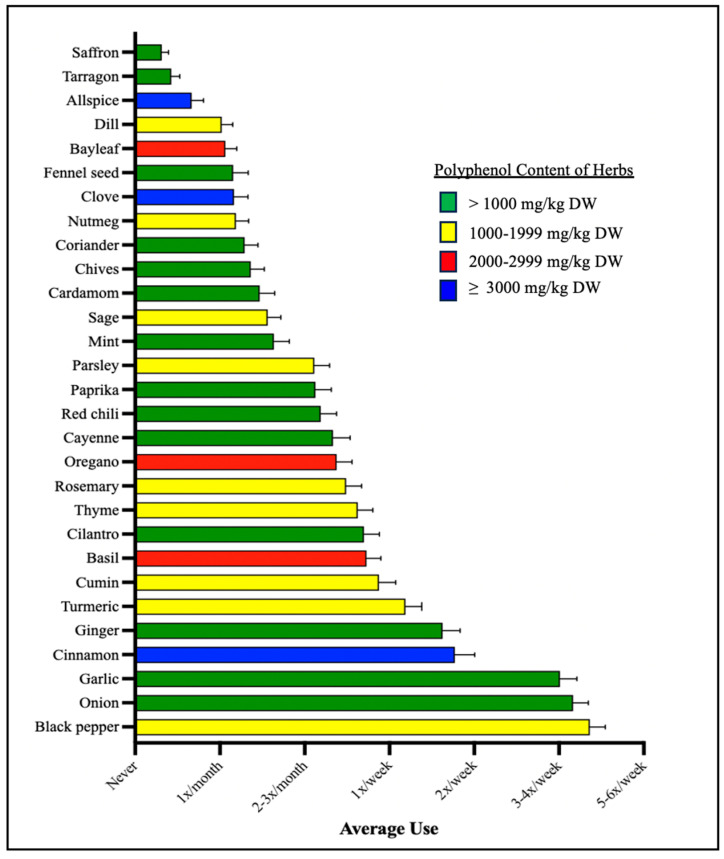
Average herb and spice use. Shown here are the average frequencies with which each herb and spice was used by study participants. Each herb and spice are color labeled by the category of its total polyphenol contents in milligrams (mg) of total polyphenols per kilogram (kg) dry weight of the herb/spice (mg/kg DW).

To calculate polyphenol-weighted frequency scores, we used the Phenol Explorer Database [1,39] to categorize herbs and spices into one of four groups based on their total polyphenol contents in milligrams of total polyphenols per kilogram dry weight of the herb/spice (mg/kg DW; Figure 1), as follows: >1000 mg/kg DW (Group 1); 1000–1999 mg/kg DW (Group 2); 2000–2999 mg/kg DW (Group 3); and ≥3000 mg/kg DW (Group 4). We calculated a weighted frequency score for each of these four groups by summing the frequency scores of herbs within each group and then multiplying that summed score by the group number. 

For example, Group 4 contains herbs with a polyphenol content of ≥3000 mg/kg DW according to the PED), and includes cinnamon, clove, and allspice; if a participant’s frequency score for each herb is 4, 0, and 7, respectively, a summed frequency score for Group 4 is created by 4 + 0 + 7 = 11. Then, a final weighted frequency score is created by multiplying 11 (the summed score) by the group number: 11 × 4 = 44. 

As the survey does not assess the actual quantity of herbs and spices consumed, only the frequency of use, the weighted frequency score accounts for the fact that certain herbs and spices may contribute more polyphenols per instance of consumption than others. 

### 2.6. Statistical Analysis 

The three gut microbial outcomes for this study include measures of alpha diversity (Shannon Index), beta diversity (Bray–Curtis dissimilarity), and microbial taxa abundance [38]. Differences in the alpha diversity (Shannon Index) between *low-*, *medium-*, and *high*-exposure groups were assessed by Kruskal–Wallis test with a post-hoc Wilcoxon Rank Sum test. A Principal Coordinate Analysis (PCoA) based on Bray–Curtis dissimilarity metrics was used to observe differences in bacterial communities between *low-*, *medium-*, and *high*-exposure groups. Differences in Bray–Curtis distances were statistically analyzed using a permutational analysis of variance (PERMANOVA) with alpha = 0.05. 

Then, relationships between microbial taxa abundance and polyphenol consumption were explored in a two-step approach. First, potential microbial biomarkers of polyphenol intake were identified by using a linear discriminate analysis (LDA) effect size (LEfSe). This LEfSe analysis assessed differences in bacterial taxa abundance between *low-*, *medium-*, and *high*-exposure groups and the effect size of those differences with a threshold LDA score of >2. As the purpose of this study was to explore microbial taxa that may be related to differing quantities of polyphenol consumption, which could be used in the subsequent targeted analysis, alpha = 0.01 for the LEfSe. Then, the microbial biomarkers identified via LEfSe were used in a Spearman’s rank correlation and a heatmap of correlation coefficients to explore relationships between microbial taxa abundance and the mg/day consumption of total polyphenols, as well as the mg/day consumption of the specific major polyphenol classes (e.g., flavonoids, phenolic acids, lignans, stilbenes, other). 

The diversity and LeFSe analyses were performed in the MicrobiomeAnalyst online platform [33], while the Spearman’s Rank analyses were performed in GraphPad Prism 10 for macOS (Version 10.0.2).

## 3. Results

### 3.1. Characteristics of Study Participants

Participant demographics, cardiometabolic measures, and substance use history (e.g., smoking history, frequency of alcohol use) are reported in Table 1. The majority of participants were white (~78%), non-Hispanic (86.5%), and female (84.4%). Cardiometabolic measures for participants were, on average, within normal physiological ranges. Additionally, the majority of participants were non-smokers (88.5%); out of the 11 participants who reported being smokers, nine reported smoking 1–3 times per month and only two reported smoking daily. Finally, around 60% of participants reported consuming alcoholic beverages from never to three times per month, with only about 6% reporting use from five times per week to daily. The distribution of these measures across the low-, medium-, and high-polyphenol consumer groups are described in Supplementary Materials (Table S1). 

#### 3.1.1. Dietary Polyphenol Consumption

The average estimated dietary polyphenol consumption is reported in Table 2. Values for these exposure variables are reported for the entire sample in addition to being stratified by *low-*, *medium-*, and *high-consumer* categories based on tertile distributions. The largest major class of polyphenols consumed by all participants on average were flavonoids, followed by phenolic acids, lignans, “other”, and stilbenes (Table 2). Flavonoids on average comprised about half of the participants’ total polyphenol consumption. 

#### 3.1.2. Herb and Spice Use

Out of the 29 herbs measured, only six herbs and spices were, on average, consumed at least once per week including black pepper, onion, garlic, cinnamon, ginger, and turmeric (Figure 1). Three out of these six herbs fell into the >1000 mg/kg DW category, as listed in the Phenol Explorer Database (PED): onion, garlic, and ginger. Black pepper, the most frequently used herb, and turmeric both fell into the 1000–1999 mg/kg DW category. Only one of these six herbs, cinnamon, fell into ≥3000 mg/kg DW category. In total, 14 of the herbs and spices used fell into the >1000 mg/kg DW category, with 9 herbs and spices in the 1000–1999 mg/kg DW category, 3 herbs and spices in the 2000–2999 mg/kg DW category, and 3 herbs and spices in the ≥3000 mg/kg DW category.

### 3.2. Microbial Community Profiling Stratified by Estimated Dietary Polyphenol Consumption Categories

**Figure 2:**
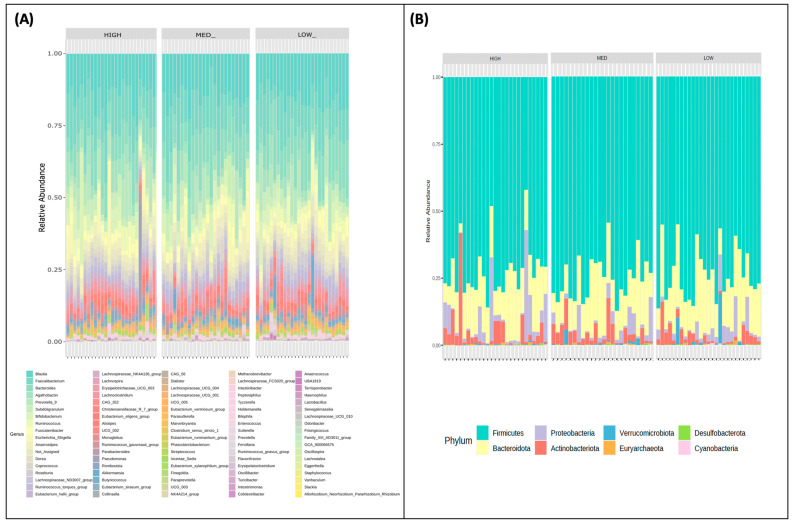
Community profiling by polyphenol consumption categories. The relative abundances of participants’ (**A**) phyla and (**B**) genera are stratified by low, medium, and high consumers of dietary polyphenols.

Relative genus (Figure 2A) and phylum-level (Figure 2B) abundance for each participant is described and stratified by exposure categories. All groups were characterized by the Firmicutes being the dominant phyla, followed by Bacteroidota, Actinobacteria, and Proteobacteria with no significant differences between groups. There also appear to be two outliers in the high group with respect to Proteobacteria abundance. 

### 3.3. Microbial Taxa Abundance, but Not Diversity, Differs by Estimated Dietary Polyphenol Consumption Categories

**Figure 3:**
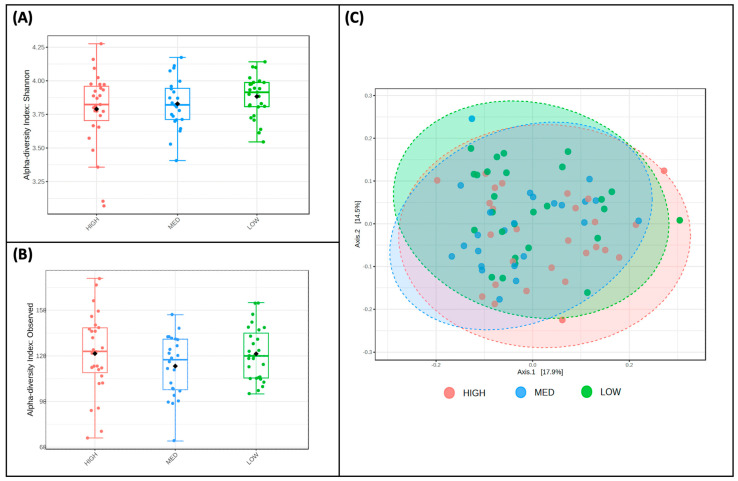
Diversity does not differ by estimated polyphenol consumption. Two measures of alpha diversity, Shannon Index (**A**) and observed richness (**B**), are stratified by low, medium, and high consumers of dietary polyphenols; no significant features were detected. Bray—Curtis dissimilarity metrics (**C**), a measure of beta diversity, are plotted between low, medium, and high consumers of dietary polyphenols; PERMANOVA revealed that no significant features were detected.

Alpha (Figure 3A,B) and beta diversity (Figure 3C) measures were described for participants and stratified by exposure categories. The Shannon Index values, a measure of alpha diversity, did not differ between low (M = 3.88, SD = 0.15), medium (M = 3.82, SD = 0.19), and high (M = 3.79, SD = 0.28) consumers of dietary polyphenols in this sample (Figure 3A). Observed richness, another measure of alpha diversity, also did not differ between low (M = 121.41, SD = 17.54), medium (M = 113.29, SD = 20.09), and high (M = 121.56, SD = 26.56) consumers of dietary polyphenols in this sample (Figure 3B). Bray–Curtis dissimilarity distances were plotted using Principal Coordinate Analysis (PCoA) plotted Bray–Curtis dissimilarity metrics of low, medium, and high consumers of dietary polyphenols (Figure 3C); PERMANOVA using these metrics revealed no differences in the beta diversity of microbial communities between these groups.

**Figure 4:**
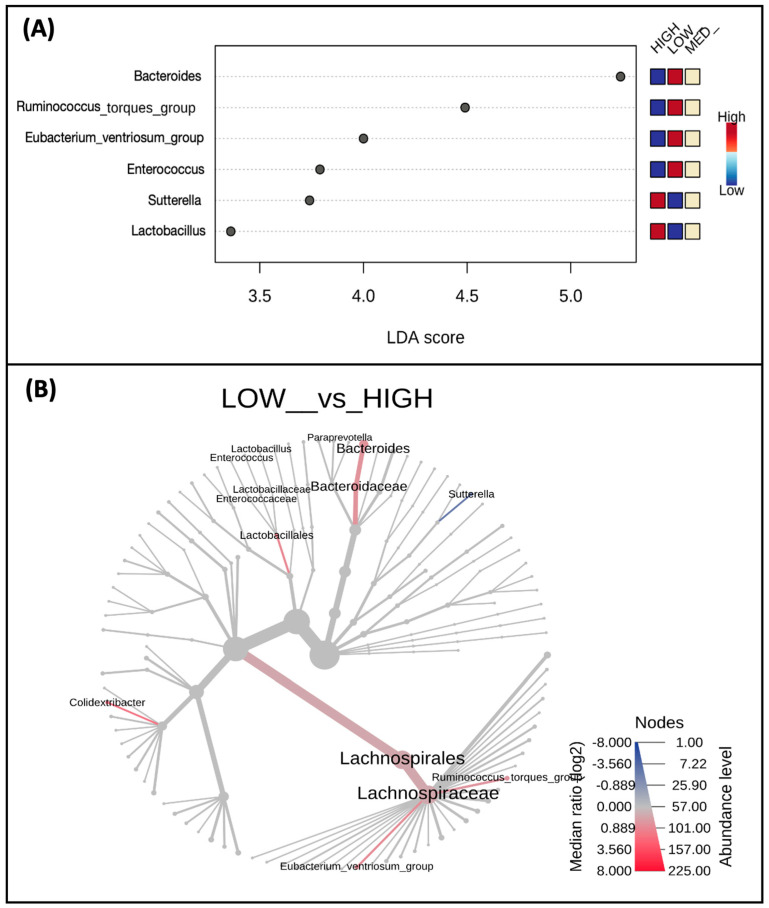
Taxa abundance differs by estimated polyphenol consumption. Shown are taxa at the genus level (**A**) which were identified as biomarkers of polyphenol consumption and differ by low, medium, and high consumers; only taxa with *p* < 0.1 are displayed. A phylogenetic heat tree (**B**) displaying a differential abundance of genera (*p*-value < 0.1) was detected by Wilcoxon Rank Sum comparing the low and high consumers.

When examining differences in taxa abundance at the genus level between low, medium, and high consumers of dietary polyphenols (Figure 4A), the abundance of *Lactobacillus* (*p*-value = 0.007) and *Sutterella* (*p*-value = 0.064) was highest in the high-consumer group and lowest in the low-consumer group. Conversely, the abundance of *Eubacterium ventriosum group* (*p*-value = 0.014), *Ruminococcus torques group* (*p*-value = 0.038), *Bacteroides* (*p*-value = 0.052), and *Enterococcus* (*p*-value = 0.057) in the low-consumer group and lowest in the high-consumer group (Figure 4A). A phylogenetic heat tree comparing abundances between the low versus high consumer groups, including differential abundances analyzed by Wilcoxon Rank Sum, are also represented (Figure 4B). 

### 3.4. Microbial Taxa Abundance, but Not Diversity, Differs by the Frequency of Polyphenol-Weighted Culinary Herb and Spice Use

**Figure 5:**
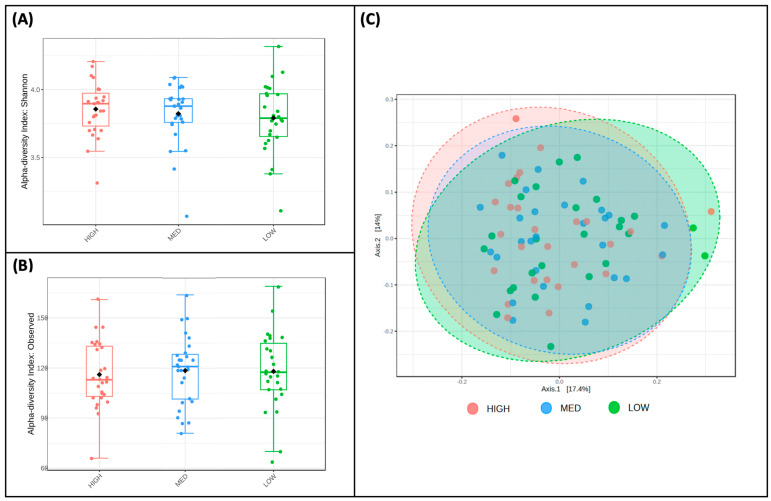
Diversity does not differ by frequency of polyphenol-weighted herb and spice use. Two measures of alpha diversity, Shannon Index (**A**) and observed richness (**B**), are stratified by low-, medium-, and high-frequency use of polyphenol-weighted herbs and spices; no significant features were detected. Bray–Curtis dissimilarity metrics (**C**), a measure of beta diversity, are plotted between low-, medium-, and high-frequency use of polyphenol-weighted herbs and spices; PERMANOVA revealed that no significant features were detected.

Alpha and beta diversity measures were for participants and stratified by exposure categories. The Shannon Index values, a measure of alpha diversity, did not differ between low-frequency (M = 3.79, SD = 0.26), medium-frequency (M = 3.82, SD = 0.22), and high-frequency (M = 3.86, SD = 0.20) users of polyphenol-containing herbs and spices (Figure 5A). Observed richness, another measure of alpha diversity, also did not differ between low (M = 118.04, SD = 23.32), medium (M = 118.57, SD = 20.54), and high (M = 116.11, SD = 19.98) consumers of dietary polyphenols in this sample (Figure 5B). Principal Coordinate Analysis (PCoA) plotted Bray–Curtis dissimilarity metrics of low-, medium-, and high-frequency users of polyphenol-containing herbs and spices (Figure 5C); PERMANOVA using these metrics revealed no differences in the beta diversity of microbial communities between these groups. 

**Figure 6:**
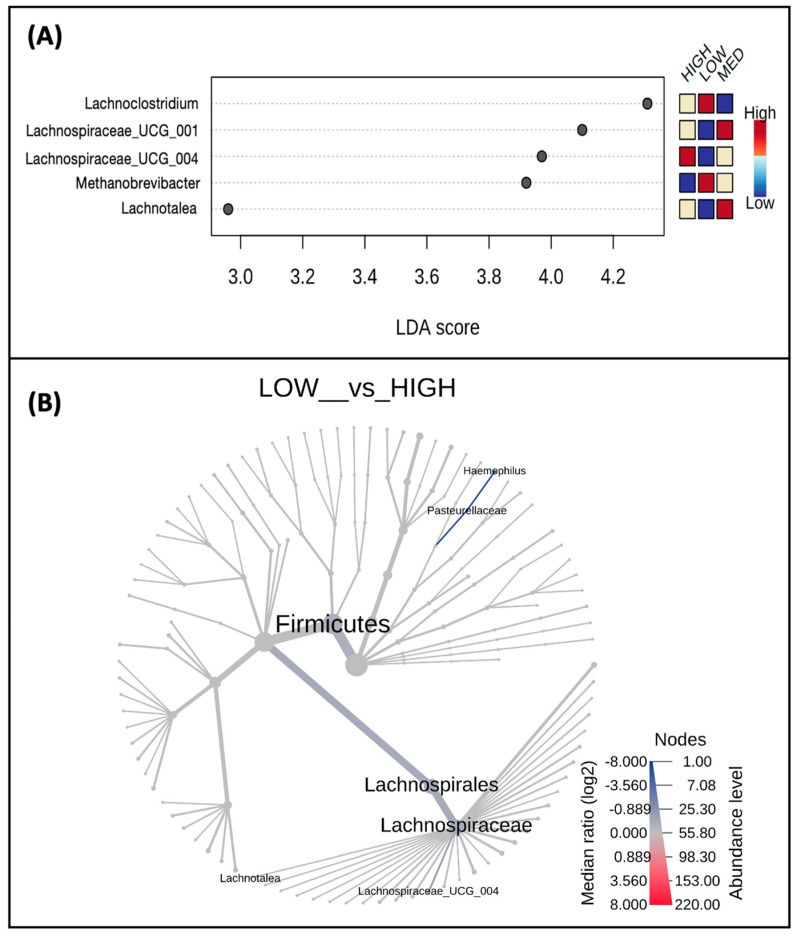
Taxa abundance differs by frequency of polyphenol-weighted herb and spice use. Shown are taxa at the genus level (**A**) which were identified as biomarkers of polyphenol-weighted herb and spice use and differ by low-, medium-, and high-frequency users; only taxa with *p* < 0.1 are displayed. A phylogenetic heat tree (**B**) displaying a differential abundance of genera (*p*-value < 0.1) was detected by Wilcoxon Rank Sum comparing the low- and high-frequency users.

When exploring differences in taxa abundance at the genus level between low-, medium-, and high-frequency users of polyphenol-weighted herbs and spices (Figure 6A), *Lachnospiraceae UCG 004* (*p*-value = 0.006), *Lachnotalea* (*p*-value = 0.037), and *Lachnospiraceae UCG 001* (*p*-value = 0.085) had the lowest abundance in the low-frequency group. While *Lachnospiraceae UCG 004* had the highest abundance in the high-frequency group, both *Lachnotalea* and *Lachnospiraceae UCG 001* had the highest abundance in the medium-frequency group. Conversely, the abundance of *Lachnoclostridium* (*p*-value = 0.025) and *Methanobrevibacter* (*p*-value = 0.092) were highest in the low-frequency group; the abundance of these genera decreased as the frequency of use increased. A phylogenetic heat tree comparing abundances between the low- and high-frequency users, whose differential abundances were analyzed by Wilcoxon Rank Sum, is also represented (Figure 6B).

### 3.5. Correlations between Microbiota and Different Polyphenol Classes

**Figure 7:**
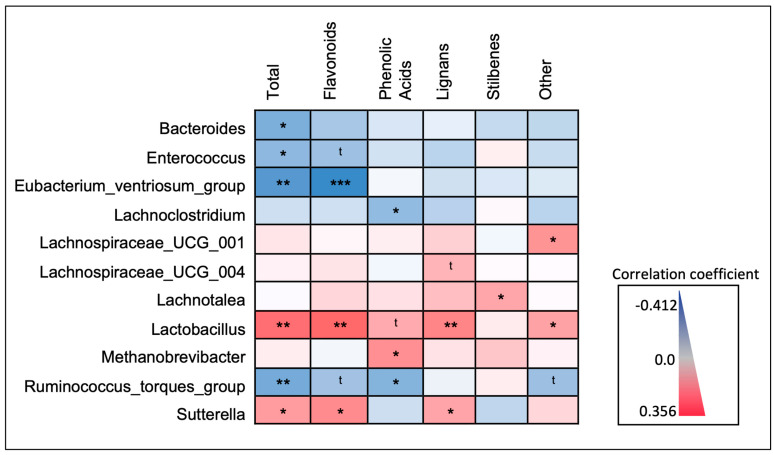
Spearman’s rank correlations between taxa abundance and estimated daily consumption of major polyphenol classes. Microbial taxa identified as biomarkers of polyphenol exposure were further explored by correlating the relative abundance of each with estimated daily polyphenol consumption (“Total”; mg/day), and the five major polyphenol classes. Shown is a heat map displaying the directionality (e.g., positive or inverse) and significance of the correlation coefficients. * *p* < 0.05; ** *p* < 0.01; *** *p* < 0.001; t < 0.1.

Relationships between microbial taxa and dietary polyphenols were further explored with Spearman’s rank correlations between relative abundance and the estimated daily consumption (mg/day) of the major classes of polyphenols (Figure 7). The directionality of relationships identified in the LEfSe model of participants stratified by total polyphenol consumption (Figure 4A) was mirrored with continuous total polyphenol consumption (mg/day), in addition to several of the major polyphenol classes (Figure 5). Both *Lactobacillus* (*p*-value = 0.002) and *Sutterella* (*p*-value = 0.031) abundance were significantly and positively correlated with estimated total daily polyphenol consumption. When looking at specific polyphenol classes, *Lactobacillus* abundance was significantly and positively correlated with daily consumption of flavonoids (*p*-value = 0.001) and lignans (*p*-value = 0.008). Similarly, the *Sutterella* abundance was also significantly and positively correlated with the estimated daily consumption of flavonoids (*p*-value = 0.013) and lignans (*p*-value = 0.046). 

The abundance of *Eubacterium ventriosum* (*p*-value = 0.001), the *Ruminococcus torques group* (*p*-value = 0.009), *Bacteroides* (*p*-value = 0.014), and *Enterococcus* (*p*-value = 0.037) were all inversely correlated with total polyphenol consumption. In addition, the *Eubacterium ventriosum group* was also significantly and inversely correlated with the daily consumption of flavonoids (*p*-value = <0.001), and the abundance of the *Ruminococcus torques group* was significantly and inversely correlated with the daily consumption of phenolic acids (*p*-value = 0.024). Both *Bacteroides* and *Enterococcus* abundance did not display any significant correlations with specific polyphenol classes, although *Enterococcus* abundance displayed an inverse trending relationship (*p*-value < 0.1) with estimated daily flavonoid consumption. 

Regarding taxa previously identified as significant in the LEfSe models of participants stratified by frequency of polyphenol-weighted herb and spice consumption (Figure 6A), some but not all relationships indicated in the LEfSe models were mirrored with consumption of the major polyphenol classes (Figure 7); none were significantly correlated with total polyphenol consumption. The abundance of *Lachnospiraceae UCG-004*, *Lachnospiraceae UCG-001*, and *Lachnotalea* was highest in either the high- or medium-frequency consumer categories. *Lachnospiraceae UCG-001* was significantly and positively correlated with the estimated daily consumption of “other” polyphenols (*p*-value = 0.020) and *Lachnotalea* was significantly and positively correlated with the estimated daily consumption of stilbenes (*p*-value = 0.049). *Lachnospiraceae UCG-004* displayed a positive trending relationship (*p*-value < 0.1) with estimated daily lignan consumption. The abundance of *Lachnoclostridium* and *Methanobrevibacter* was highest in the low-frequency consumer categories. While both *Lachnoclostridium* (*p*-value = 0.049) and *Methanobrevibacter* (*p*-value = 0.015) abundance was significantly correlated with phenolic acid consumption, *Lachnoclostridium* displayed a negative correlation while *Methanobrevibacter* displayed a positive correlation. A full correlation matrix with correlation coefficients, 95% CI’s, and *p*-values is available in Supplementary Materials (Table S2).

## 4. Discussion

This study aimed to explore relationships between gut microbial measures and habitual dietary polyphenol consumption in a sample of healthy adults. Although primarily examining estimated dietary polyphenol consumption in the context of food and beverage sources, we secondarily sought to explore relationships between the frequency of polyphenol-weighted herb and spice consumption and gut microbial measures as well. In the case of both exposures, differential trends in microbial taxa abundance but not alpha or beta diversity measures were observed between low-, medium-, and high-exposure groups, while the literature regarding the effect of dietary polyphenol consumption on gut microbial diversity is conflicting and may be polyphenol and/or metabolite specific [43,44,45]. 

### 4.1. Microbiota Observed to Be Positively Correlated with Dietary Polyphenols

Previous studies examining changes in microbial taxa abundance in response to polyphenol-based interventions are not consistent, and an increase or decrease in abundance of specific taxa appears to differ with dietary source and/or dose of polyphenol, as well as pathophysiological state (e.g., healthy vs. specific disease state). However, the relationship between estimated dietary polyphenol consumption and the abundance of several microbial taxa observed in this study reflects the findings of some existing literature. For example, we observed that individuals with higher estimated total polyphenol, flavonoid, and lignan consumption also had a higher abundance of *Lactobacillus* and *Sutterella.* This trend was previously described for both taxa in response to a flavonoid-enriched apple intervention in healthy adults [46]; for *Sutterella* abundance in response to lignan-rich interventions (e.g., flaxseed meal [47,48] and psyllium husk [49]; and for *Lactobacillus* abundance in response to other flavonoid-rich interventions [14,50]. There is previous evidence that flavonoids promote *Lactobacillus* growth [51], and several *Lactobacillus* spp., are capable of metabolizing both flavonoids and lignans [52,53]. It is important to note that other studies observed either no change in *Lactobacillus* abundance [54] or a decrease in *Sutterella* abundance [55]. 

The microbial taxa *Lachnospiraceae UCG-004*, *Lachnospiraceae UCG-001*, and *Lachnotalea* were identified as possible biomarkers of polyphenol-weighted herb and spice use, displaying the highest abundance in either the high- or medium-frequency user group. However, only *Lachnospiraceae UCG-001* and *Lachnotalea* displayed a weak positive correlation with consumption of the other polyphenols and stilbenes, respectively. Little has been described regarding the effects of polyphenol intake on *Lachnospiraceae UCG-001* and *Lachnospiraceae UCG-004*, except some in vitro and in vivo research [56,57], although polyphenol-based interventions in clinical trials have noted increases in other *Lachnospiraceae* [54,55,56]. Likewise, there is very limited research noting changes in *Lachnotalea* abundance [58,59] and *Methanobrevibacter* abundance [60,61] in response to polyphenol and/or herb and spice interventions. 

Regarding their relevance in health and disease, little is known about the role of *Lachnotalea*, and while specific beneficial effects have not been widely described for *Sutterella*, its role as a commensal versus pathogenic bacteria may be species specific [62]. However, several of these other taxa are known to facilitate the regulation of immune, cardiometabolic, and intestinal barrier function-related processes through multiple mechanisms. *Methanobrevibacter* [63] may play a role in cardiovascular health through the depletion of trimethylamine (TMA), a precursor to the cardiovascular risk factor trimethylamine oxide (TMAO), for methanogenesis [64], and was inversely associated with obesity and BMI [65,66,67] and, separately, with serum TMAO levels [68]. 

*Lactobacillus* and *Lachnospiraceae* are producers of short-chain fatty acids (SCFA; [69,70]) and are involved in the transformation of bile acids [70,71], both of which are important immunoregulatory [72,73] and cardiometabolic signaling molecules [74,75]. These molecules regulate immune responses locally (i.e., at the intestinal mucosa) and systemically, subsequently supporting barrier integrity and anti-inflammatory activities [76,77], in addition to modulating both glucose [78,79] and lipid metabolism [76,80]. *Lactobacillus* also play a key role in maintaining intestinal health through several other mechanisms [81,82,83,84,85,86,87,88]. While *Lactobacillus* and *Lachnospiraceae* promote beneficial regulatory processes under homeostatic conditions, their overabundance has also been noted in certain systemic autoimmune diseases (e.g., rheumatoid arthritis, systemic lupus erythematosus, Primary Sjogren’s Syndrome [89,90,91]). Additionally, there are conflicting results regarding *Lactobacillus* overgrowth or depletion in cardiometabolic [92,93,94] and gastrointestinal conditions [92,95], which also may be disease specific. 

### 4.2. Microbiota Observed to Be Inversely Correlated with Dietary Polyphenols

Some microbial taxa in this study displayed an inverse relationship with total estimated polyphenol consumption, including *Eubacterium ventriosum group*, *Ruminococcus torques group*, *Bacteroides*, and *Enterococcus*, and several previous studies corroborate these inverse relationships.

A dietary intervention using varying concentrations of fruit- and vegetable-derived flavonoids demonstrated an inverse relationship between flavonoid intake and *Bacteroides* abundance [96] and, separately, the use of other polyphenol-based interventions (e.g., red wine polyphenol [14], cranberry [12]) also noted decreases in *Bacteroides* abundance. Red wine is rich in flavonoids [1,39], and interestingly, the study that observed a decrease in *Bacteroides* abundance in response to a red wine polyphenol intervention also noted increases in *Lactobacillus* [14]; this trend was also observed in our own study. However, *Bacteroides* abundance was also observed to increase in response to certain polyphenol-based interventions [97,98]. Additionally, while several in vivo and in vitro studies using mixed [99] or flavonoid-rich [50] interventions noted decreases in *Enterococcus* [100,101,102], studies in human populations observed increases in *Enterococcus* abundance in conjunction with increases in *Lactobacillus*. These differences may be influenced by the type of polyphenol used, as some *Bacteroides* spp. and *Enterococcus* spp. are capable of metabolizing certain types of flavonoids [103].

Both *Ruminococcus torques* and *Eubacterium ventriosum groups* were not only inversely correlated with estimated total polyphenols, but also phenolic acid consumption and flavonoid consumption, respectively. For the *Ruminococcus torques group*, the inverse relationship with phenolic acid consumption and inverse trend with the consumption of flavonoids and “other” polyphenols are reflected in clinical and in vivo studies that use flavonoid-based [104,105], phenolic acid-based [106], and fermented vegetable juice [107] interventions. While some *Eubacterium* spp. are capable of metabolizing certain flavonoids [103], it is unclear if this extends to the *Eubacterium ventriosum group* specifically. Finally, *Lachnoclostridium*, was inversely correlated with phenolic acid consumption. While we did not identify phenolic acid-specific responses in the existing literature, inverse relationships were observed between *Lachnoclostridium* and other polyphenols in vitro [51] and in vivo [108]. 

Many of these bacteria are involved in the production of clinically relevant immunoregulatory molecules, such as short-chain fatty acids (e.g., *Lachnoclostridum*, [69,109], *Bacteroides* [110], *Eubacterium ventriosum* [111]) and bile acids (e.g., *Bacteroides*, *Enterococcus* [70,112]), and can play an important role in host nutrition availability [113]. However, several are also well-known opportunistic pathogens (e.g., *Enterococcus* spp. [114,115], *Bacteroides* spp. [116]) and/or contain qualities, such as the mucolytic activity of *Ruminococcus torques group* [117], which may have implications for gastrointestinal health and inflammation [118]. Additionally, several of these taxa (e.g., *Ruminococcus torques group*, *Lachnoclostridium*, *Eubacterium ventriosum*) have been implicated in the presence of cardiovascular disease risk factors for obesity [119], obesity [120], and associated anthropometrics (e.g., visceral fat, BMI [121,122]), with evidence also highlighting a role for *Lachnoclostridum* in the biosynthesis of trimethylamine (TMA), a precursor to the cardiovascular disease biomarker trimethylamine oxide (TMAO) [122]. There also appears to be a common thread with some of these taxa being abundant in various nephropathies, either being correlated with disease outcomes (e.g., *Lachnoclostridium*, *Bacteroides* and *Ruminococcus torques group* [123]) or their increased abundance observed in diseased individuals (e.g., *Enterococcus*, *Eubacterium ventriosum group*, and *Lachnoclostridium* [124]). 

### 4.3. Strengths and Limitations

One notable strength is that, unlike many other studies that have investigated the response of microbiota to polyphenol-based interventions, the present research investigated these relationships in habitual diet and incorporated habitual culinary herb and spice consumption. As these data were from a sample of generally healthy adults absent of certain physiological factors (e.g., cardiometabolic risk factors) that may be associated with shifts in gut microbiota, these relationships between microbiota and polyphenol consumption may be generalizable to the wider healthy US population. Additionally, this study also supports the validity of an innovative algorithm for calculating the estimated polyphenol consumption from VioScreen dietary analysis data. As many of the relationships observed in our Spearman’s rank correlations between microbial taxa and individual classes mirror findings from previous interventional and/or mechanistic studies, we can be confident in the estimations provided. It is worth noting that the observed mean polyphenol consumption in this sample also reflects average values of US adults (n = 9773), as reported in NHANES data from five surveys spanning 2007–2016 [125]. These observations support the sample’s dietary polyphenol consumption as being representative of the general population. 

There are also several limitations of this study. This study is somewhat limited in addressing the contribution of polyphenols from culinary herb and spice sources, as the herb and spice questionnaire only provided data on the frequency of use, not on the quantity consumed. It is also important to note that certain factors that may be associated with gut microbiota (e.g., supplement usage, medication usage, smoking, alcohol consumption, biological sex) were not included in statistical analyses, as the subgroups of these factors were of vastly unequal sample sizes. Moreover, these data are from a relatively small sample of healthy adults in a specific geographical location. While the relationships observed in our Spearman’s rank correlations between microbial taxa and individual classes indicate that these results are may be generalizable to the larger population of healthy adults (i.e., several relationships mirror findings from previous interventional and/or mechanistic studies), growing this study to include a larger sample size and multiple geographical locations would aid in its generalizability and translatability.

## 5. Conclusions

- In this study, we observed that microbial taxa, but not microbial diversity measures, differed by levels of daily polyphenol consumption from dietary and herb and spice sources in generally healthy US adults.
- Our results suggest that higher quantities of habitual polyphenol consumption may support an intestinal environment where opportunistic and pathogenic bacteria are represented in a lower relative abundance compared to those with less potentially virulent qualities.
- These findings, particularly correlations between microbiota and daily consumptions of specific polyphenol classes, may have implications for the development of precision polyphenolic interventions for microbiota targets, as well as dietary guidelines for polyphenolic intake.
- Future directions of implementing this investigation on a larger scale across different geographical regions would help build a larger reference base for microbial biomarkers of polyphenol exposure in healthy US adults. This framework could be used to investigate the relationships between habitual polyphenol consumption and gut microbiota in specific disease populations to examine how these microbial biomarkers of polyphenol exposure may differ in individuals already experiencing specific pathologies or dysbiosis.

## Figures and Tables

**Table 1:** Characteristics of study participants (n = 96).

Variables	Value
**Age**	***M*** **(*SD*)**
	29.3 (6.1)
**Sex Assigned at Birth**	**n (%)**
Male	14 (14.6)
Female	81 (84.4)
Intersex	1 (>1)
**Race**	**n (%)**
White/Caucasian	75 (78.1)
Asian	5 (5.2)
African American	2 (2)
Middle Eastern	2 (2)
Native Hawaiian/Pacific Islander	1 (1)
American/Alaska Native	1 (1)
Mixed	6 (6.3)
Other/Unknown	4 (4.2)
**Ethnicity**	**n (%)**
Hispanic/LatinX	9 (9.4)
Non-Hispanic/LatinX	83 (86.5)
Unknown	4 (4.2)
**Cardiometabolic Measures**	***M*** **(*SD*)**
BMI (kg/m^2^)	23.7 (4.3)
Triglycerides (mg/dl)	88.5 (47.0)
Total cholesterol (mg/dl)	170 (29.1)
LDL (mg/dl)	73.4 (33.8)
HDL (mg/dl)	62.2 (21.4)
Systolic blood pressure (mmHg)	113.6 (12.2)
Diastolic blood pressure (mmHg)	65.2 (9.1)
Hemoglobin A1c (%)	4.3 (0.6)
**Smoking History**	**n (%)**
Smoker	11 (11.5)
Non-smoker	85 (88.5%)
**Alcohol Use Frequency**	**n (%)**
Never	21 (21.9)
1-3x/month	37 (38.5)
1-2x/week	19 (19.8)
3-4x/week	13 (13.5)
5-6x/week	4 (4.2)
Daily	2 (2.1)

Characteristics of study participants. Shown are demographic, cardiometabolic, and substance use history of study participants (n = 96). *M* = mean; *SD* = standard deviation; n = count; % = percent of sample. BMI = body mass index; LDL = low-density lipoprotein; HDL = high-density lipoprotein.

**Table 2:** Estimated dietary polyphenol exposure of study participants (n=96).

**Estimated Dietary Polyphenol Intake (mg/day)**				
**Consumer Category**	***All***	***Low***	***Med***	***High***
Total Polyphenols	1224.11 (661.67)	557.64 (198.68) (n = 34)	1131.27 (169.97) (n = 32)	1986.42 (467.70) (n = 30)
Flavonoids	590.23 (343.00)	270.86 (80.89) (n = 34)	504.37 (77.61) (n = 32)	998.24 (258.16) (n = 30)
Phenolic Acids	487.54 (445.01)	117.16 (53.24) (n = 34)	363.62 (105.28) (n = 32)	985.83 (430.02) (n = 30)
Lignans	105.86 (80.79)	36.19 (12.66) (n = 34)	85.16 (15.43) (n = 32)	196.12 (77.21) (n = 30)
Stilbenes	0.90 (1.47)	0.03 (0.04) (n = 34)	0.38 (0.14) (n = 32)	2.31 (1.89) (n = 30)
Other	23.16 (15.65)	9.60 (3.79) (n = 35)	20.08 (2.97) (n = 30)	40.45 (14.78) (n = 31)
**Weighted Frequency Score**				
**Frequency Category**	***All***	***Low***	***Med***	***High***
Average Frequency Score	102.93 (19.27)	59.75 (19.99) (n = 33)	96.81 (8.19) (n = 28)	153.14 (29.61) (n = 35)

Estimated dietary polyphenol exposure of study participants. Shown here are the mean estimated dietary polyphenol intake values (mg/day) for study participants. The mean value (M) and standard deviation (SD) are shown for all participants (*All*), as well as those stratified into *low-*, *medium*- (*Med*), and *high*-exposure groups (n = population).

## Data Availability

The data presented in this study are available within this article.

## Supplementary Materials

The following supporting information can be downloaded at: https://www.mdpi.com/article/10.3390/nu16060773/s1, Table S1: Distribution of substance use history, cardiometabolic measures, age, and sex across all participants (*All*), as well as those stratified into *Low*, Medium (*Med*), and *High* polyphenol consumer groups; (n = population; % = percentage of n; M = mean; SD = standard deviation). Table S2: Full data for correlations between microbiota and polyphenol classes. Shown here are the Spearman’s rho (*r*) correlation coefficients, *p*-values, and 95% confidence intervals (CI) for correlations between microbiota and daily con-sumption (mg/kg dry weight) of total polyphenols and the major polyphenol classes.
