# Supplementary material for: Relationships between Habitual Polyphenol Consumption and Gut Microbiota in the INCLD Health Cohort
Source: Nutrients. 2024 Mar 8;16(6):773. doi: 10.3390/nu16060773 (PMC10974568; doi:10.3390/nu16060773)
Supplement: Supplementary file 1 [file nutrients-16-00773-s001.zip › nutrients-2862216-supplementary.pdf]

nutrients

Table S1. Distribution of substance use history, cardiometabolic measures, age, and sex across all participants (*All*), as well as those stratified into *Low*, Medium (*Med*), and *High* polyphenol consumer groups; (n = population; % = percentage of n; M = mean; SD = standard deviation).

Variables	*All* (*n* = 96)	*Low* (*n* = 34)	*Med* (*n* = 32)	*High* (*n* = 30)
**Age**	***M (SD)***	***M (SD)***	***M (SD)***	***M (SD)***
	29.3 (6.1)	29.7 (8.8)	30.8 (6.5)	29.4 (5.8)
**Sex Assigned at Birth**	***n (%)***	***n (%)***	***n (%)***	***n (%)***
Male	14 (14.6)	2 (5.9%)	8 (25%)	4 (13.3%)
Female	81 (84.4)	32 (94.1%)	24 (75%)	25 (83.3%)
Intersex	1 (>1)	0	0	1 (3.3%)
**Cardiometabolic Measures**	***M (SD)***	***M (SD)***	***M (SD)***	***M (SD)***
BMI (kg/m^2)	23.7 (4.3)	21.6 (9.7)	22.2 (6.9)	21.9 (8.8)
Triglycerides (mg/dl)	88.5 (47.0)	84.4 (33.4)	80.4 (35.9)	98.8 (65.3)
Total Cholesterol (mg/dl)	170 (29.1)	179.3 (30.4)	169.6 (26.4)	158.1 (26.4)
LDL (mg/dl)	73.4 (33.8)	74.3 (38.6)	68.7 (31.9)	76.4 (27.9)
HDL (mg/dl)	62.2 (21.4)	69.6 (21.6)	66.4 (25.3)	50.6 (13.2)
Systolic blood pressure (mmHg)	113.6 (12.2)	110.8 (7.5)	116.5 (11.6)	112.9 (15.2)
Diastolic blood pressure (mmHg)	65.2 (9.1)	65.9 (7.4)	64.4 (8.8)	64.4 (10.6)
Hemoglobin A1c (%)	4.3 (0.6)	4.3 (0.7)	4.2 (0.6)	4.3 (0.7)
**Smoking History**	***n (%)***	***n (%)***	***n (%)***	***n (%)***
Smoker	11 (11.5%)	3 (8.8%)	5 (15.6%)	3 (10%)
Non-smoker	85 (88.5%)	31 (91.2%)	27 (84.4%)	27 (90%)
**Alcohol Use Frequency**	***n (%)***	***n (%)***	***n (%)***	***n (%)***
Never	21 (21.9)	10 (29.4%)	5 (15.6%)	6 (20%)
1-3x/month	37 (38.5)	15 (44.1%)	12 (37.5%)	11 (36.7%)
1-2x/week	19 (19.8)	6 (17.6%)	6 (18.8%)	7 (23.3%)
3-4x/week	13 (13.5)	3 (8.8%)	5 (15.6%)	5 (16.7%)
5-6x/week	4 (4.2)	0	2 (6%)	2 (6.7%)
Daily	2 (2.1)	0	2 (6%)	0

Table S2. Full data for correlations between microbiota and polyphenol classes. Shown here are the Spearman's rho (*r*) correlation coefficients, *p*-values, and 95% confidence intervals (CI) for correlations between microbiota and daily consumption (mg/kg dry weight) of total polyphenols and the major polyphenol classes.

	**Total Polyphenols**	**Flavonoids**	**Phenolic Acids**	**Lignans**	**Stilbenes**	**Other**
	R<br>[95% CI]<br>(*p*-value)	R<br>[95% CI]<br>(*p*-value)	R<br>[95% CI]<br>(*p*-value)	R<br>[95% CI]<br>(*p*-value)	R<br>[95% CI]<br>(*p*-value)	R<br>[95% CI]<br>(*p*-value)
Bacteroides	−0.281<br>[−0.476, −0.051]<br>(0.123)	−0.169<br>[−0.393, 0.051]<br>(0.140)	−0.087<br>[−0.287, 0.169]<br>(0.451)	−0.047<br>[−0.252, 0.206]<br>(0.682)	−0.083<br>[−0.331, 0.122]<br>(0.469)	−0.117<br>[−0.341, 0.111]<br>(0.309)
Enterococcus	−0.251<br>[−0.442, −0.008]<br>(0.026)	−0.214<br>−0.410, 0.030]<br>(0.059)	−0.088<br>[−0.300, 0.155]<br>(0.447)	−0.132<br>[−0.348, 0.102]<br>(0.248)	0.075<br>[−0.170, 0.287]<br>(0.517)	−0.086<br>[−0.322, 0.131]<br>(0.456)
Eubacterium_ ventriosum_gr.	−0.243<br>[−0.542, −0.140]<br>(0.032)	−0.329<br>[−0.586, −0.202]<br>(0.003)	0.049<br>[−0.218, 0.240]<br>(0.670)	−0.042<br>[−0.305, 0.149]<br>(0.712)	−0.048<br>[−0.284, 0.171]<br>(0.674)	0.042<br>[−0.277, 0.180]<br>(0.715)
Lachnoclostridium	−0.033<br>[−0.311, 0.143]<br>(0.722)	−0.107<br>[−0.310, 0.145]<br>(0.351)	−0.130<br>[−0.431, 0.006]<br>(0.257)	−0.088<br>[−0.354, 0.096]<br>(0.442)	0.096<br>[−0.197, 0.260]<br>(0.404)	−0.110<br>[−0.349, 0.102]<br>(0.337)
Lachnospiraceae_ UCG_001	0.103<br>[−0.149, 0.306]<br>(0.371)	0.066<br>[−0.187, 0.270]<br>(0.564)	0.078<br>[−0.168, 0.288]<br>(0.497)	0.203<br>[−0.010, 0.350]<br>(0.074)	0.050<br>[−0.006, 0.430]<br>(0.664)	0.251<br>[0.037, 0.465]<br>(0.027)
Lachnospiraceae_ UCG_004	0.147<br>[−0.177, 0.280]<br>(0.198)	0.153<br>[−0.144, 0.310]<br>(0.182)	0.091<br>[−0.225, 0.233]<br>(0.428)	0.226<br>[−0.036, 0.405]<br>(0.047)	0.047<br>[−0.197, 0.260]<br>(0.683)	0.088<br>[−0.200, 0.258]<br>(0.443)
Lachnotalea	−0.008<br>[−0.204, 0.254]<br>(0.943)	0.090<br>[−0.113, 0.339]<br>(0.435)	0.043<br>[−0.138, 0.316]<br>(0.708)	0.205<br>[−0.062, 0.384]<br>(0.071)	0.163<br>[−0.006, 0.430]<br>(0.153)	0.014<br>[−0.197, 0.260]<br>(0.905)
Lactobacillus	0.242<br>[0.127, 0.533]<br>(0.033)	0.305<br>[0.138, 0.541]<br>(0.007)	0.188<br>[−0.016, 0.422]<br>(0.099)	0.235<br>[0.076, 0.495]<br>(0.039)	0.048<br>[−0.159, 0.296]<br>0.679)	0.150<br>[0.004, 0.438]<br>(0.191)
Methanobrevibacter	0.053<br>[−0.167, 0.289]<br>(0.643)	−0.004<br>[−0.224, 0.234]<br>(0.970)	0.264<br>[0.049, 0.474]<br>(0.019)	0.058<br>[−0.143, 0.311]<br>(0.612)	0.190<br>[−0.080, 0.368]<br>(0.095)	0.012<br>[−0.178, 0.279]<br>(0.919)
Ruminococcus_ torques_gr.	−0.269<br>[−0.489, −0.069]<br>(0.017)	−0.190<br>[−0.407, 0.034]<br>(0.096)	−0.274<br>[−0.457, −0.028]<br>(0.015)	−0.029<br>[−0.241, 0.217]<br>(0.801)	0.059<br>[−0.166, 0.290]<br>(0.604)	−0.194<br>[−0.417, 0.022]<br>(0.089)
Sutterella	0.250<br>[0.017, 0.449]<br>(0.027)	0.281<br>[0.054, 0.478]<br>(0.013)	−0.113<br>[−0.315, 0.139]<br>(0.324)	0.251<br>[−0.002, 0.434]<br>(0.027)	−0.118<br>[−0.342, 0.109]<br>(0.305)	0.135<br>[−0.117, 0.336]<br>(0.237)
